# A data-driven mathematical model for evaluating the societal and economic burden of delayed access to innovative medicines

**DOI:** 10.3934/publichealth.2025036

**Published:** 2025-07-03

**Authors:** Foteini Theiakou, Catherine Kastanioti, Dimitris Rekkas, Nikolaos Kontodimopoulos, Dimitris Zavras

**Affiliations:** 1 Department of Business Administration and Organizations, University of Peloponnese, Kalamata, Greece; 2 Department of Pharmacy, University of Athens, Athens, Greece; 3 Department of Economics and Sustainable Development, Harokopio University, Athens, Greece; 4 Department of Public Health Policy, University of West Attica, Athens, Greece

**Keywords:** pharmaceutical policy, innovative medicines, social loss, reimbursement delays, mathematical model, data-quality

## Abstract

**Background:**

This study presents a novel mathematical framework to quantify the societal and economic impacts of delays in the reimbursement and distribution of innovative medicines.

**Methods:**

Utilizing the concept of years of life lost (YLL) as a measure of premature mortality, the framework calculated the impact of delay on YLL, years of potential productive life lost (YPPLL), and cost of productivity loss (CPL). The proposed model incorporated mortality probabilities through the Heligman-Pollard (HP) model, examining how delays influence health outcomes, particularly for patients awaiting treatments like Icosapent ethyl.

**Results:**

The findings reveal that extended delays significantly increase mortality and economic losses, emphasizing the need for timely access to high-value therapies. This mathematical framework not only emphasizes the adverse effects of delayed reimbursement on populations but also highlights the importance of high-quality data in accurately assessing these effects. By ensuring completeness, consistency, and reliability in healthcare data, the framework advocates for evidence-based policy decisions that promote equitable healthcare access and minimize disparities.

**Conclusions:**

The present study underscores the importance of efficient pharmaceutical policymaking in maximizing the societal benefits of innovative treatments, ensuring that both health outcomes and economic sustainability are prioritized in healthcare systems.

## 1. Introduction

Pharmaceutical policy refers to a set of laws, regulations, and strategies designed to manage the development, distribution, and use of medicines within a healthcare system. Its primary objective is to ensure that the population has access to safe, effective, and affordable medicines, while also promoting rational drug use and ensuring sustainability in healthcare costs. Pharmaceutical policy supports decision-making about how society can use its available resources to improve health and reduce inequalities.

A well-structured pharmaceutical policy typically includes several key components, such as drug approval and safety regulation. A regulatory framework ensures that all drugs meet established standards and are safe for public consumption through regulatory agencies [1] and pricing and reimbursement policies to manage drug costs and ensure equitable access to essential medicines [2]. However, formulating pharmaceutical policy for innovative medicines presents significant challenges, particularly in balancing the introduction of new, high-cost treatments with maintaining fair competition and ensuring equitable access to novel treatments, as these often represent significant advancements over existing therapies, offering more effective, safer, or personalized options for managing diseases [3],[4]. According to a study by the World Health Organization [1], introducing new medications has significantly reduced mortality rates for conditions such as cardiovascular diseases and cancers, highlighting their importance in extending life expectancy.

Furthermore, by preventing disability and premature death, innovative medicines help preserve human capital [5]. A recent study demonstrated that early intervention with innovative treatments in diseases such as rheumatoid arthritis and multiple sclerosis can prevent long-term disability, allowing individuals to maintain active and fulfilling lives [6]. This indicates that innovative medicines are not only vital for individual health but also for economic productivity. A report by the European Federation of Pharmaceutical Industries and Associations (EFPIA) found that employees with access to effective treatments for conditions like diabetes and depression showed improved productivity and reduced sick leave [7]. A study by Claxton et al. [8] examined the impact of delayed access to innovative medicines in the United Kingdom, demonstrating how even modest delays can lead to significant losses in quality-adjusted life years (QALYs), thereby impacting overall societal welfare.

However, the approval of a treatment does not necessarily translate into immediate availability of novel therapy for all patients, as delays in the reimbursement process decision can significantly hinder access, particularly in publicly funded healthcare systems [9]. Such delay can prolong patient suffering, lead to worsening health conditions, and widen health inequalities, especially for those who cannot afford to pay out-of-pocket for expensive therapies [4]. On the other hand, innovative medicines are not associated with a confirmed clinical benefit [10], so reimbursement procedures require a series of time-consuming steps by the HTA authorities [11].

Data accuracy, completeness, and reliability influence the decision-making process at multiple levels, from clinical trials to post-market surveillance [12]. Without robust data governance, the evaluation of innovative drugs becomes challenging, potentially leading to delays in market entry, pricing inefficiencies, and risks to patient safety [13]. In the context of innovative drug development, data quality plays a pivotal role in clinical research, particularly in randomized controlled trials (RCTs) and real-world evidence (RWE) studies. Regulatory agencies, such as the U.S. Food and Drug Administration (FDA) and the European Medicines Agency (EMA), increasingly rely on high-quality RWE to complement RCTs in assessing the safety and effectiveness of novel therapies [13]. Poor data quality in these studies can lead to biased outcomes and misinterpretation of efficacy results, ultimately hindering patient access to groundbreaking treatments [13].

Pharmaceutical policy is deeply influenced by data integrity in health technology assessments (HTAs), which guide drug pricing and reimbursement decisions. Incomplete or inconsistent data may result in inaccurate cost-effectiveness evaluations, affecting the affordability and accessibility of innovative drugs [14]. Furthermore, the integration of big data and artificial intelligence in drug discovery and regulatory decision-making necessitates stringent data validation processes to prevent algorithmic biases and ensure equitable healthcare access [14]. To optimize pharmaceutical policy outcomes, stakeholders must prioritize investments in data infrastructure, interoperability, and regulatory frameworks that enhance data reliability. Strengthening data governance will enable more efficient drug approval processes, fairer pricing models, and better patient outcomes, ultimately fostering an ecosystem that supports pharmaceutical innovation while maintaining safety and cost-effectiveness.

While the existing literature extensively addresses the clinical benefits of innovative medicines [4] and the importance of timely access [10],[11], few studies provide a quantifiable, data-driven framework that estimates the cumulative societal and economic burden specifically associated with delays in the reimbursement process decisions. Most analyses to date focus either on isolated health outcomes (e.g., QALYs) or macro-level health system indicators, without dynamically linking delays to population-level metrics such as years of life lost (YLL), years of potential productive life lost (YPPLL), and cost of productivity loss (CPL). To address this gap, we propose a novel mathematical model that incorporates age-specific mortality risks via the Heligman-Pollard model [15] with economic productivity metrics grounded in the human capital approach [16],[17]. This approach allows for a holistic assessment of the broader consequences of delayed access, encompassing both health loss and productivity loss. Furthermore, while some studies have explored productivity implications of illness or premature death [17], they have not explicitly modeled delay as a modifiable health policy variable. Therefore, our study offers a novel perspective for pharmaceutical policy evaluation—one that is mathematically rigorous, data-informed, and policy-relevant.

The objective of the study is to outline a novel mathematical framework regarding the delayed entry of novel medicines as an independent variable affecting (a) years of life loss (YLL) [18] and (b) years of potential productive life loss (YPPLL), where YPPLL refers to the productive years an individual would have lived in the absence of an event as well as the cost of productivity loss (CPL) [19]. By factoring in the age at death, rather than just the occurrence of death, this calculation aims to provide a more accurate representation of the societal burden or impact of a particular cause of mortality. Its main purpose is to assess the relative significance of various causes of early death within a population, helping health planners prioritize prevention efforts. Additionally, the proposed mathematical framework offers a guidance tool for policy-makers to evaluate the broader impact of new medicines. However, the reliability and accuracy of such evaluations are inherently dependent on high-quality data, as inconsistencies or biases in data inputs can lead to misleading policy decisions. Ensuring robust data governance and validation mechanisms enhances the framework's ability to generate meaningful insights for pharmaceutical policy-making. Ultimately, ensuring high data quality strengthens the foundation of pharmaceutical policies, leading to improved healthcare efficiency, cost-effectiveness, and better patient outcomes.

## 2. Materials and methods

The years of life lost (YLL) is a widely used public health measure to assess premature mortality. YLL is a metric used to assess premature mortality by considering both how often deaths occur and the age at which they happen; as such, it estimates the time a person would have lived if they had not died prematurely. YLL for a particular cause is calculated by multiplying the number of deaths (Ν) due to that cause by a loss function (LF) that defines the years lost based on the age of death.

The basic formula for YLL, for a specific cause (c), age (a), sex (s), and year (t), as reported by WHO, is as follows [20]:

$$\begin{array}{c} Years\;of\;life\;lost\equiv YLL=f\left(cause,age,\text{sex},time\;period\right)=\\ N\left(cause,age,\text{sex},time\;period\right)L\left(age,sex\right) \end{array}$$ (1)

where:

N:Number of deaths caused by a cause c

at a specific age x for sex s during a time period t,

L≡Loss function=f(age,sex)

Brustugun [18] expressed the YLL measure as follows:

$$YLL=\overset{X}{\underset{x=0}{\sum }}\left(number\;of\;deaths\;at\;age\;x\right)\left(expected\;remaining\;life\;years\;at\;age\;x\right)$$ (2)

To quantify the societal impact of delayed access to innovative medicines, it is necessary to express the number of deaths as a function of age, based on function (2). To achieve this, a calculation of the probability (mortality rate) *q_x_*, which represents the likelihood that a person at age *x* will die within a year, was performed:

$$q_{x}=\frac{d_{x}}{l_{x}}$$ (3)

where:

*d_x_* is the number of deaths at age *x*, and *l_x_* is the number of people alive at age *x*.

It is important to note that *d_x_* is the number of people who died before reaching age *x* + 1, thus we can write that *d_x_* = *l_x_* − *l_x_*_+1_, i.e., the difference of people alive between *age x* + *and age x*.

The Heligman-Pollard (HP) model [15] is given by:

$$q_{x}=[A^{{\left(x+B\right)}^{C}}+De^{(-E{\left(lnx-lnF\right)}^{2}}+\frac{GH^{x}}{1+GH^{x}}]$$ (4)

where A, B, C, D, E, F, G, H are parameters to be estimated (4) and measure the probability *q_x_* that a person of age *x* will die before age *x* + 1.

Since equations (3) and (4) express the same measure, i.e., *q_x_* as shown in equation (5) below,

$$q_{x}=[A^{{\left(x+B\right)}^{C}}+De^{(-E{\left(lnx-lnF\right)}^{2}}+\frac{GH^{x}}{1+GH^{x}}]=\frac{d_{x}}{l_{x}}$$ (5)

*d_x_* could then be calculated as follows:

$$d_{x}=l_{x}[A^{{\left(x+B\right)}^{C}}+De^{(-E{\left(lnx-lnF\right)}^{2}}+\frac{GH^{x}}{1+GH^{x}}]$$ (6)

The HP model illustrates the variation in mortality rates across different stages of life, from childhood and young adulthood to old age. The first term of equation (4) captures mortality trends in early life: parameter A represents the child mortality rate, B reflects the mortality rate at age one, and C indicates the decline in mortality over time (ages 0–9). The second term addresses the increase in mortality due to accidents in adulthood and maternal deaths among women of reproductive age (ages 10–40), with D representing the severity, E indicating the concentration, and F denoting the peak position of this “hump”. The third term follows Gompertz's law, where G represents the baseline mortality level for older adults, and H indicates its rate of increase beyond age 40 [21],[22].

The proposed framework suggests the following equation (7) where the dependent variable *q_x_* depends on the variable *x* that is defined as follows:

x=age of diagnosis+delay (7)

The following proposed mathematical model quantifies the number of deaths:

$$d_{x}=l_{x}[A^{[(age\;of\;diagnosis+delay)+B){]}^{C}}+De^{{\left(-E\left[\ln\left(age\;of\;diagnosis+delay\right)-lnF\right)\right]}^{2}}+\frac{GH^{\left(age\;of\;diagnosis+delay\right)}}{1+GH^{\left(age\;of\;diagnosis+delay\right)}}]$$ (8)

The variable *delay* is defined as the number of days between the marketing authorization of a novel medicine and its reimbursement approval (i.e., reimbursement delay) [23]. However, in equation (7), the variable *delay* is defined as the difference between the patient's age at diagnosis and their age at death without access to the treatment. This assumption reflects a real-world scenario in which a patient is diagnosed with a serious condition but does not survive long enough to benefit from the treatment due to a delay in access. Therefore, in this context, *delay* serves as a proxy for the lost opportunity window, emphasizing the clinical and societal consequences of delayed access.

The mathematical model assumes that there is no “delay” on the day of diagnosis. However, for patients who were diagnosed and died within the same year, the “delay” is the same across all cases.

Thus, after substituting *x* = *age_diagnosis_* + *delay* into equation (2), *YLL* is expressed as a function of the variable *delay*:

YLL=∑x=0X(lx[A[(age of diagnosis+delay)+B)]C+De(−E[ln(age of diagnosis+delay)−lnF)]2+GH(age of diagnosis+delay)1+GH(age of diagnosis+delay)])(expected remaining life years at age x) (9)

However, health policy affects the variable *delay*:

$$\begin{array}{l} delay=f(health\;policy)\\ =\{\begin{array}{ll} delay, & when\;health\;policy=0,\;meaning\;that\;a\;health\;policy\;does\;not\;exist\\ 0, & health\;policy=1,\;meaning\;that\;a\;health\;policy\;exists \end{array} \end{array}$$ (10)

Then, equation 9 can be revised as flows:

$$\begin{array}{l} YLL\\ =\overset{X}{\underset{x=0}{\sum }}(l_{x}[A^{{\left[\left(age\;of\;diagnosis+f\left(health\;policy\right)+B\right)\right]}^{C}}\\ +De^{{\left(-E\left[\ln\left(age\;of\;diagnosis+f(health\;policy\right)-lnF\right)\right]}^{2}}\\ +\frac{GH^{\left(age\;of\;diagnosis+f(health\;policy\right)}}{1+GH^{\left(age\;of\;diagnosis+f(health\;policy\right)}}])(expected\;remaining\;life\;years\;at\;age\;x) \end{array}$$ (11)

According to the proposed mathematical framework, the variable *delay* significantly influences the YLL measure, thereby impacting not only individual patient outcomes but also broader societal and economic aspects.

In addition to the YLL measure, delayed access to novel medicines is also considered to impact productivity, in terms of the years of potential productive life lost (YPPLL) measure [20]. The YPPLL measure projects the economic and social impacts of an event due to premature death, which are public health priorities for policymaking. It is widely recognized that premature mortality not only affects individual health outcomes but also imposes significant economic burdens on society by reducing workforce participation and productivity [20]. It is worth noting that the human capital approach should be employed to compute both permanent and temporary losses resulting from premature mortality and absenteeism, respectively. This method quantifies economic losses by estimating the present value of future earnings lost due to death or disability, making it a valuable tool for assessing the broader economic implications of public health interventions [18]. It is worth noting that the human capital approach should be employed to compute permanent and temporary losses that are due to premature mortality and absenteeism, respectively [22].

The basic formula for the YPPLL measure is written as follows:

$$YPPLL=\overset{J}{\underset{j}{\sum }}number\;of\;deaths_{j}(retirement\;age-mean\;age_{j})$$ (12)

where *j is the* age cohorts.

After substituting the number of deaths in equation 12, it could be written as follows:

$$\begin{array}{l} YPPLL=\overset{J}{\underset{j}{\sum }}(l_{j}[A^{[(age\;of\;diagnosis+delay)+B){]}^{C}}+De^{{\left(-E\left[\ln\left(age\;of\;diagnosis+delay\right)-lnF\right)\right]}^{2}}\\ +\frac{GH^{\left(age\;of\;diagnosis+delay\right)}}{1+GH^{\left(age\;of\;diagnosis+delay\right)}}])(retirement\;age-mean\;age_{j}) \end{array}$$ (13)

Productivity loss is a measure of the resources lost when employees work at suboptimal levels or are absent from work. It encompasses both absenteeism (missed workdays) and presenteeism (reduced efficiency while at work), both of which have significant economic implications for employers and society [21]. A societal perspective, which aims at maximizing overall welfare, commonly incorporates all relevant costs, including productivity losses due to disease-related impairments [21]. This perspective ensures that healthcare interventions are evaluated not only in terms of direct medical costs but also in terms of their broader economic consequences, such as lost earnings and reduced labor force participation. The cost of productivity loss (CPL) is often estimated using the human capital approach, which calculates the present value of lost earnings due to illness or premature mortality, or the friction cost method, which accounts for the time required to replace a worker and restore productivity levels [22].

The general formula for the cost of productivity loss (CPL) is as follows:

$$CPL=\underset{J}{\sum }YPPLL_{j}\;GDP\;per\;capita$$ (14)

$$\begin{array}{l} CPL=\overset{J}{\underset{j}{\sum }}(l_{j}[A^{[(age\;of\;diagnosis+delay)+B){]}^{C}}+De^{{\left(-E\left[\ln\left(age\;of\;diagnosis+delay\right)-lnF\right)\right]}^{2}}\\ +\frac{GH^{\left(age\;of\;diagnosis+delay\right)}}{1+GH^{\left(age\;of\;diagnosis+delay\right)}}])(retirement\;age-mean\;age_{j})GDP\;per\;capita \end{array}$$ (15)

**Figure 1.**
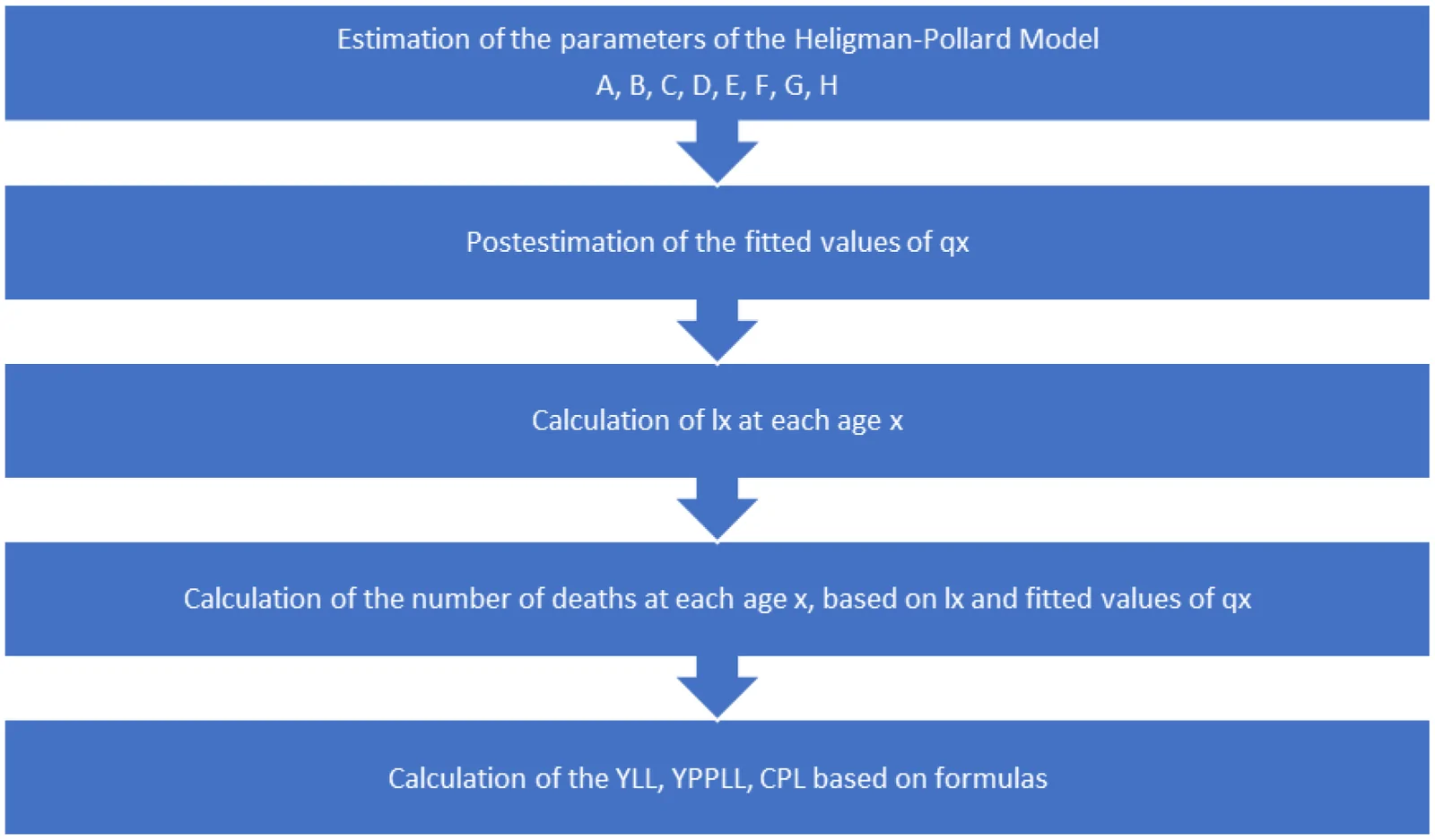
Methodology's flowchart.

Subsequently, the proposed mathematical model was applied to a dataset related to the novel drug Icosapent ethyl to estimate YLL, YPPLL, and CPL (see Figure 1). By integrating these key metrics, our mathematical framework provides a comprehensive assessment of the broader societal and economic consequences associated with delayed access to innovative treatments. This approach not only quantifies the direct health burden in terms of premature mortality but also captures the long-term economic implications of lost workforce productivity, reinforcing the critical need for timely pharmaceutical policy interventions.

Icosapent ethyl is a highly purified and stable ethyl ester of EPA (eicosapentaenoic acid) that has been shown to effectively reduce triglyceride levels, making it a key therapeutic option for addressing residual cardiovascular risk in patients with hypertriglyceridemia [23]. It is approved as an adjunct to diet for adult patients with triglyceride levels of at least 500 mg/dL, a condition associated with an increased risk of pancreatitis and cardiovascular disease. Unlike other omega-3 fatty acid formulations, Icosapent ethyl has demonstrated unique cardioprotective properties beyond triglyceride lowering, likely due to its anti-inflammatory, anti-thrombotic, and membrane-stabilizing effects.

According to the results of the REDUCE-IT trial [24], treatment with Icosapent ethyl led to a 25% relative risk reduction in major cardiovascular events compared to placebo in patients with elevated triglyceride levels, despite concurrent statin therapy. The trial included 8179 patients who were either 45 years or older with established cardiovascular disease or 50 years or older with diabetes mellitus and at least one additional cardiovascular risk factor. Eligible patients also had a fasting triglyceride level of at least 135 mg/dL and an LDL-C level between 41 and 100 mg/dL, ensuring that the observed effects were independent of standard lipid-lowering therapy. The primary endpoint was a composite of cardiovascular death, nonfatal myocardial infarction, nonfatal stroke, coronary revascularization, or unstable angina, analyzed in a time-to-event framework. Additionally, the secondary endpoint evaluated cardiovascular death, nonfatal myocardial infarction, and nonfatal stroke, reinforcing the robustness of the observed clinical benefit.

The selection of Icosapent ethyl in this study is justified by its strong evidence-based cardiovascular benefits and potential to prevent productivity loss by reducing premature mortality and morbidity associated with cardiovascular disease. Given that cardiovascular diseases are among the leading causes of YLL and YPPLL globally [25],[26], the timely incorporation of Icosapent ethyl into clinical practice and pharmaceutical policy can yield substantial societal and economic benefits.

Although the Heligman-Pollard model is typically applied to mortality data across the entire age range (0 ≤ x < ∞) [15], it has also been utilized for specific age groups, such as individuals aged 60+ and 85+ years [27]. Moreover, it has been employed to analyze cause-specific mortality rates [27]. Therefore, it is well-suited for examining CVD-related mortality in individuals aged 45–86 years.

In line with the study objective, data from the REDUCE-IT trial were analyzed using the MortalityLaws package of R [28].

To calculate YLL, YPPLL, and CPL measures, estimation was performed for the eight parameters of the Heligman-Pollard model, with 95% confidence intervals and p-values obtained by bootstrapping, using the *x and q_x_* values from the aforementioned trial. Assuming an initial cohort *l_x_* = 160,000, we calculated the number of deaths via the calculation of *fitted values of q_x_* via the following equation:

$$number\;of\;deaths_{x}=l_{x}\;fitted\;values\;of\;q_{x}$$ (16)

while

$$l_{x+1}=l_{x}-l_{x}\;fitted\;values\;of\;q_{x}$$ (17)

However, since the number of data points was n = 15,000 (daily age measurements starting from 0, corresponding to 45 years), only the four quarters of each age were retained. This approach ensured consistency with sample size requirements while aligning with the fact that the Heligman-Pollard model primarily applies to annual data. The Heligman-Pollard model is a parametric mortality model fitted by optimizing a predefined loss function aimed at minimizing estimation error. The *MortalityLaws* R package provides the implementation of eight distinct loss functions, enabling better capture across different segments of the mortality distribution. Thus, estimation is achieved via optimization of one of the eight predefined loss functions.

In this paper, we do not discuss the machine learning (ML) approaches; rather, we use a different mathematical data-driven model, i.e., the Heligman-Pollard model. According to Heligman and Pollard [15], a law of mortality, i.e., a parameterization function, is a mathematical expression for the graduation of the age pattern of mortality that fits mortality data. In this sense, the term “data-driven mathematical model” in the paper's title is consistent with the methodology and analysis of the model we used, even though, in our days, the term usually refers to ML.

For the calculation of *YPPLL* and *CPL*, ages were grouped into the following five-year intervals: (1) 45–49 years, (2) 50–54 years, (3) 55–59 years, (4) 60–64 years, (5) 65–69 years, (6) 70–74 years, (7) 75–79 years, and (8) 80–84 years. Ages 85–86 were excluded due to the lack of available data for forming the 85–89 age group. However, for estimating the *fitted values of q_x_*, we used the entire age range of 45–86 years. The retirement age was set at 67 years. The deviance goodness-of-fit test was applied for model evaluation, and Microsoft Excel was used for the calculation of YLL, YPPLL and CPL.

## 3. Data sources and ethical considerations

The proposed model applied to a cohort of 15,000 observations based on the inclusion criteria of the REDUCE-IT trial, targeting individuals aged 45–86. The dataset was adjusted to reflect population-specific mortality dynamics within the 45–86 age range, excluding patients not eligible for Icosapent ethyl treatment. No direct patient-level data was used as data derived from the REDUCE-IT clinical trial [24]. The original dataset mentioned above included 15,000 observations across daily age measurements starting from 45 years. Assuming a cohort l_x = 160,000, we calculated the outcome measures (YLL, CPL, YPPLL). Given that this is a secondary analysis of publicly available trial outcomes, individual consent was not required.

## 4. Results

To evaluate the impact of delayed access to innovative medicines, the Heligman-Pollard model was applied to a dataset derived from the REDUCE-IT trial. The model's parameters were estimated to quantify the mortality dynamics within the studied population and to compute key metrics such as YLL, YPPLL, and CPL. The bootstrapping method was employed to generate 95% confidence intervals and p-values, ensuring statistical robustness.

Based on the analysis, the eight parameters of the Heligman-Pollard model were found to be A = 0.0003, B = 0.0040, C = 0.2149, D = 0.0010, E = 10.1647, F = 16.4506, G = 0.0030, and H = 1.0629.

Table 1 presents the bootstrap 95% confidence intervals and p-values for these estimates.

**Table 1.** Bootstrap 95% confidence intervals and p values.

Parameter	Estimate	p-value	95% confidence interval
A	3.2484e−04	0.0202	1.7775e−05, 3.5395e−04
B	4.0001e−03	<0.0010	4.0000e−03, 4.0010e−03
C	2.1487e−01	0.9990	1.7902e−01, 1.3872e+02
D	9.9836e−04	<0.0010	9.8522e−04, 1.0000e−03
E	1.0164e+01	<0.0010	1.0101e+01, 1.1644e+01
F	1.6450e+01	<0.0010	1.2564e+01, 1.6654e+01
G	2.9952e−03	0.0131	1.3749e−03, 6.0150e−03
H	1.0629e+00	<0.0010	1.0524e+00, 1.0749e+00

As shown in Table 1, only the parameter *C* is not statistically significant.

The deviance goodness-of-fit test indicated a good fit of the model (deviance = 2.3439, df = 157, p = 1). Notably, *l_x_* tends to 0 at the age of 72 years. By substituting the number of deaths at *age_x_* and *the expected remaining life years at age_x_* in equation (2):

$$YLL=\overset{X}{\underset{x=0}{\sum }}\left(number\;of\;deaths\;at\;age\;x\right)\left(expected\;remaining\;life\;years\;at\;age\;x\right)$$ (18)

*YLL* was calculated, yielding a total of 5,266,149.101 years.

Then, considering the age cohorts *j*: 1) 45–49, 2) 50–54, 3) 55–59, 4) 50–64, 5) 65–69, 6) 70–74, 7) 75–79, and 8) 80–84) in equation (10):

$$YPPLL=\underset{j}{\sum }number\;of\;deaths_{j}(retirement\;age-mean\;age_{j})$$ (19)

*YPPLL* was estimated to yield a total of 2,783,568 years.

Finally, using *the calculated YPPLL and the GDP per capita* (17,347 €), in equation (11):

$$CPL=\underset{J}{\sum }YPPLL_{j}\;GDP\;per\;capita$$ (20)

*CPL* was amounted to 48,286,550,895 €.

**Figure 2.**
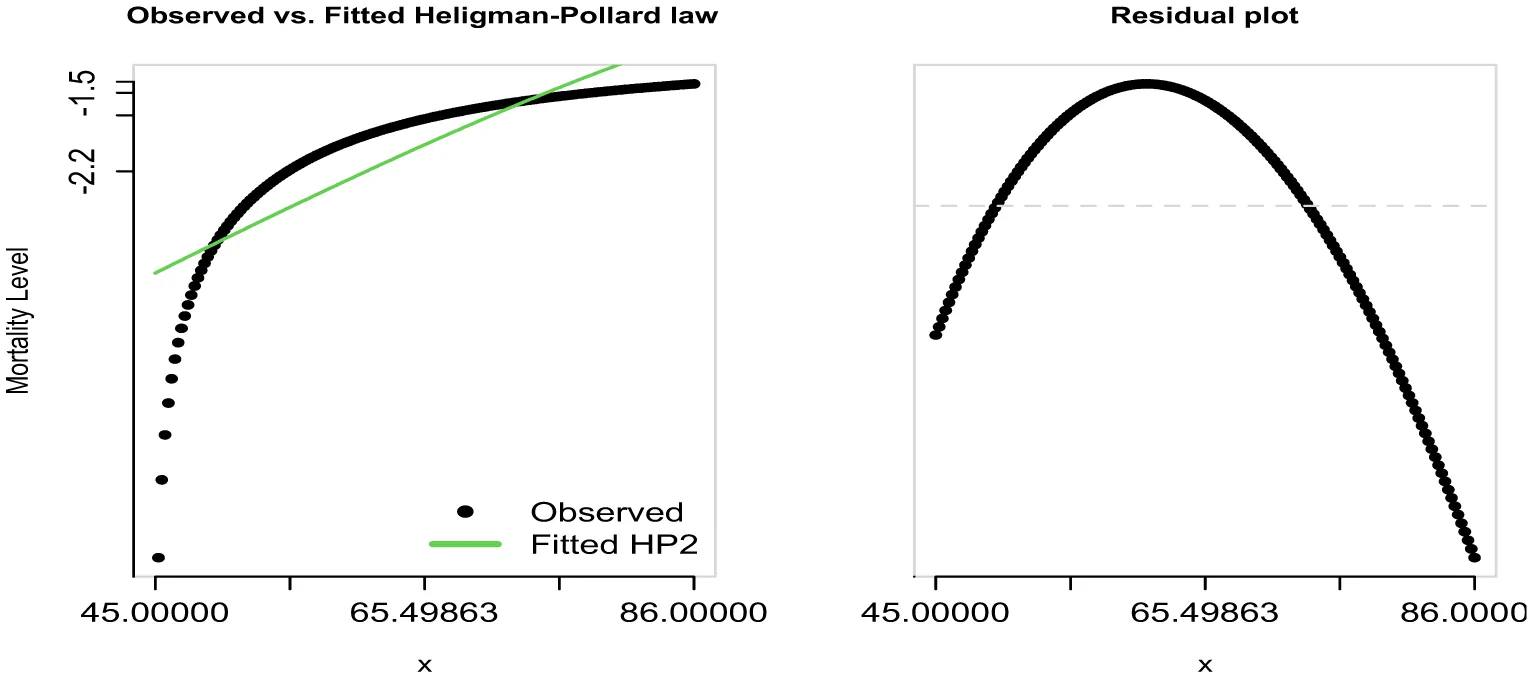
Model fit of the Heligman-Pollard curve to age-specific mortality data (ages 45–84).

Figure 2 demonstrates an adequate fit of the model to the observed data; however, this goodness of fit is conditioned by the exclusion of age groups below 45 and above 84, due to data unavailability.

## 5. Discussion

The proposed mathematical framework quantifies the societal impact of the parameter *delay*, which is influenced by pharmaceutical policymaking in terms of YLL, deaths, CPL, and YPPLL. The model indicates that a potentially highly innovative drug requires swift handling and minimal delays in the reimbursement process to avoid significant losses in deaths, YLL, YPPLL, and CPL. Conversely, when the variable *delay* is high, only therapies with limited clinical benefits should be affected. Delay in access to innovative medicines can lead to prolonged disease burden, increased healthcare costs, and reduced productivity, underscoring the critical role of timely policy interventions in minimizing public health and economic repercussions. Delayed medicine availability can result in prolonged disease burden, increased disability-adjusted life years (DALYs), and reduced workforce participation, ultimately impacting economic productivity and societal well-being [29]. Research has shown that timely access to effective treatments can significantly reduce premature mortality and improve work capacity, reinforcing the importance of timely pharmaceutical policies [30].

The results highlight the substantial societal and economic burden associated with delayed access to Icosapent ethyl, emphasizing its impact on premature mortality and productivity loss. The YLL of 5,266,149 years underscores the significant reduction in lifespan due to cardiovascular-related mortality, reflecting the urgency of timely interventions to prevent avoidable deaths. Furthermore, the YPPLL of 2,783,568 years demonstrates the profound effect of premature mortality on workforce participation, indicating that a considerable portion of the economically active population is lost before reaching retirement age. This loss translates into a substantial economic burden, as evidenced by the CPL amounting to €48.3 billion, reflecting the financial impact of reduced labor force contributions and lost economic output. These findings reinforce the critical role of pharmaceutical policy in ensuring timely access to innovative therapies, as delays in drug availability not only compromise public health but also generate significant economic costs for society. Addressing these delays through data-driven health policies and efficient regulatory frameworks can help minimize preventable deaths, improve productivity, and reduce the broader economic impact associated with untreated or undertreated cardiovascular conditions.

Moreover, the present study demonstrated that the Heligman-Pollard model effectively captured the mortality dynamics within the studied population. The estimated parameters provided a robust framework for evaluating the impact of delayed access to Icosapent ethyl on YLL, YPPLL, and CPL. The results indicated that *delays* in drug availability contribute significantly to premature mortality and economic burden, reinforcing the importance of timely pharmaceutical policy interventions. Furthermore, the bootstrap-derived 95% confidence intervals and p-values confirmed the statistical reliability of the model estimates. Given these results, integrating data-driven modeling approaches into pharmaceutical policy decision-making could enhance the evaluation of the societal and economic impact of medicine delays.

Using the mathematical model based on age of diagnosis and delay can significantly enhance decision-making regarding a specific drug or class of drugs, particularly in areas such as risk-benefit analysis, treatment guidelines, and drug approval processes. By incorporating these variables, healthcare decision-makers can refine drug treatment strategies, ensuring they target the most appropriate patient groups and optimize the benefit/risk ratio for each group. This approach leads to more precise decision-making, ultimately improving patient outcomes while managing healthcare resources more efficiently. For instance, in patients diagnosed at age 45, the model may predict a significant reduction in long-term cardiovascular mortality through early intervention. The model suggests that early intervention yields the greatest long-term benefits, highlighting the importance of timely access to innovative treatments.

Moreover, this mathematical framework emphasizes the long-term, dynamic effects of delayed access that traditional models may miss. Delayed treatment can lead to irreversible health deterioration, potentially increasing future healthcare costs and making conditions more challenging or impossible to treat effectively. It also underscores the importance of innovation itself, recognizing that earlier access not only benefits current patients but also accelerates future medical breakthroughs and enhances long-term efficiencies within the healthcare system. By factoring in these extended consequences, the framework presents a more robust analysis for policymakers, urging them to value the long-term societal and economic impacts of innovation alongside immediate healthcare outcomes.

The implications of data quality extend beyond immediate health and economic outcomes, influencing broader aspects of healthcare equity, policy effectiveness, and clinical decision-making. Given that coverage is a key domain in assessing data quality [31], the absence of data for these populations represents a significant gap for health policymakers. Additionally, the presence of inconsistent data formats across different excluded age groups further complicates policy decisions, as data format uniformity is another crucial factor in data quality assessment. Consequently, policymaking may become disproportionately focused on certain age demographics, overlooking the broader public health impact of CVDs across all age groups. Especially for CVDs, which, according to international literature, affect individuals across the entire lifespan—from early childhood to advanced age—health policymakers must consider the full spectrum of disease impact when formulating solutions to mitigate its severe societal consequences [32]. Epidemiological studies indicate that while CVD risk increases significantly with age, early-life factors such as childhood obesity, hypertension, and genetic predispositions contribute to long-term cardiovascular outcomes, reinforcing the need for comprehensive, age-inclusive health policies [33]. Since high-quality data directly contributes to informed decision-making and advancements in healthcare [8], greater emphasis should be placed on a comprehensive research design that ensures inclusivity across all relevant age groups. Consequently, to develop effective health policies, decision-makers must have access to a complete and representative dataset that captures the full spectrum of CVD incidence.

Additionally, the model emphasizes the need for a balanced approach to pharmaceutical policy—one that values both innovation and sustainability. Delays in access to innovative medicines disproportionately affect vulnerable populations, especially those in lower-income brackets or regions with limited healthcare infrastructure [8]. These groups often depend more on public healthcare systems and may lack the financial means to afford high-cost therapies out of pocket. As the model highlights, delays in the reimbursement process exacerbate health inequalities, allowing wealthier individuals to access treatments sooner, while disadvantaged populations face longer waiting times. By quantifying the social losses associated with these delays, the framework makes a compelling case for prioritizing equitable access to innovative medicines, ensuring that all patients, regardless of socioeconomic status, can benefit from the latest advancements in medical care.

While introducing innovative medicines is crucial for improving patient outcomes and advancing public health, it is equally important to manage healthcare spending to ensure the long-term viability of the health system. The framework encourages policymakers to carefully weigh the trade-offs between rapid access and economic constraints. By understanding the full scope of productivity losses, social well-being, and cost savings associated with new treatments, healthcare decision-makers can better navigate these challenges, promoting policies that maximize societal benefits without compromising the financial stability of health systems.

Some limitations to this study should be considered. Some complex healthcare variables may be oversimplified, potentially overlooking variations in real-world applications. The model assumes constant values for factors like the marginal willingness to pay for QALYs, which can vary over time and across contexts. Additionally, the model might not adequately account for external influences on healthcare access. Furthermore, the findings may not be universally applicable due to variations in healthcare systems, and the static nature of the model does not account for dynamic changes in medical technology and policy. While the broader societal benefits of timely access to innovative medicines may extend beyond individual patients, the applicability of this model is limited to the population for whom the medicine is indicated and has demonstrated clinical benefit. Therefore, the evaluation model applies only to those patients meeting the inclusion criteria of the REDUCE-IT trial. Finally, it may not fully capture the long-term consequences of delayed access to health systems or address ethical considerations surrounding equitable access to innovative medicines.

## 6. Conclusions

This study introduces an innovative mathematical framework aimed at quantifying the societal cost of *delay* in the reimbursement and distribution of novel medicines. By integrating key variables such as YLL, YPPLL, and the broader implications of productivity loss, the model offers a comprehensive perspective on the societal costs tied to delayed access to innovative treatments. The results highlight the critical importance of timely pharmaceutical policy-making, especially for innovative medications that exhibit high clinical value. Delays not only diminish the therapeutic benefits available to patients but also exacerbate economic repercussions through reduced productivity and heightened healthcare expenditures. Ultimately, this model serves as a vital decision-making instrument for policymakers, aiding in the balancing act between the urgent need for access to innovative therapies and the imperative of sustainable healthcare spending. By adopting a more holistic societal perspective, the framework facilitates a thorough evaluation of new treatments, ensuring that the benefits of medical innovation are maximized for patients, healthcare systems, and society. This methodology not only measures the immediate health burden through premature mortality but also accounts for the extended economic effects of reduced workforce productivity, emphasizing the urgent necessity for prompt and effective pharmaceutical policy actions. Also, the results highlight the critical role of data quality in ensuring accurate assessments of health and economic outcomes, demonstrating that incomplete or inconsistent data can lead to biased estimations, misinformed policy decisions, and disparities in healthcare access.

## Use of AI tools declaration

The authors declare they have not used Artificial Intelligence (AI) tools in the creation of this article.
